# Unicornuate Uterus with a Non-Communicating Rudimentary Horn: Challenges and Management of a Rare Pregnancy

**DOI:** 10.7759/cureus.40666

**Published:** 2023-06-19

**Authors:** Jyotsna Garapati, Shubhada Jajoo, Sakshi Sharma, Srinidhi Cherukuri

**Affiliations:** 1 Obstetrics and Gynaecology, Jawaharlal Nehru Medical College, Datta Meghe Institute of Higher Education & Research, Wardha, IND

**Keywords:** early identification, mullerian anomalies, obstetric outcomes, pregnancy, rudimentary horn, unicornuate uterus

## Abstract

We present a unique case of pregnancy developed in a unicornuate uterus with a non-communicating rudimentary horn, emphasizing the importance of early identification, careful management, and counseling. Our patient, a 28-year-old woman, presented with abdominal pain and premature rupture of membranes at 37 weeks of gestation. She had a history of one previous normal vaginal delivery and no significant medical or genetic factors. An emergency cesarean section was performed, and a baby boy weighing 2900 grams was delivered without complications. The uterine anomaly was identified as a unicornuate uterus with a non-communicating rudimentary horn. This case report highlights the challenges and risks associated with unicornuate pregnancies, such as fetal growth restriction and preterm labor. Timely identification, meticulous monitoring, and comprehensive counseling are crucial for optimal outcomes in such cases. Further research and larger-scale studies are needed to enhance our understanding of these rare and complex pregnancies.

## Introduction

Congenital uterine malformation occurs in approximately 1% to 10% of the general population, 2% to 8% of women experiencing infertility, and 5% to 30% of women with a history of miscarriage. This condition arises from abnormal Mullerian duct development, fusion, or absorption [1]. The variations in these prevalence rates are likely attributed to the utilization of different diagnostic methods with varying accuracy and the adoption of diverse classification systems to define these abnormalities.

Nevertheless, accurately determining the exact incidence of Mullerian tract anomalies is challenging because many subtypes allow for normal reproduction, and some women with these anomalies may choose not to have children [2].

A unicornuate uterus with a rudimentary horn is an anomaly caused by the incomplete fusion of one of the paired Mullerian ducts. It is observed in only 0.1% of the general population [3]. This condition may involve the presence of an underdeveloped or rudimentary horn, with the uterus possibly having communication and an endometrium-lined cavity. Maternal mortality is estimated to be between 6% and 23% [3]. This Mullerian anomaly carries significant obstetrical risks, including first- and second-trimester miscarriages, abnormal fetal positioning, restricted fetal growth, premature delivery, and rupture of the rudimentary horn. More than 50% of pregnancies associated with this anomaly have resulted in a rupture of the pregnant uterus, typically occurring during the third trimester and believed to be due to reduced muscle mass [4].

## Case presentation

**Figure 1:**
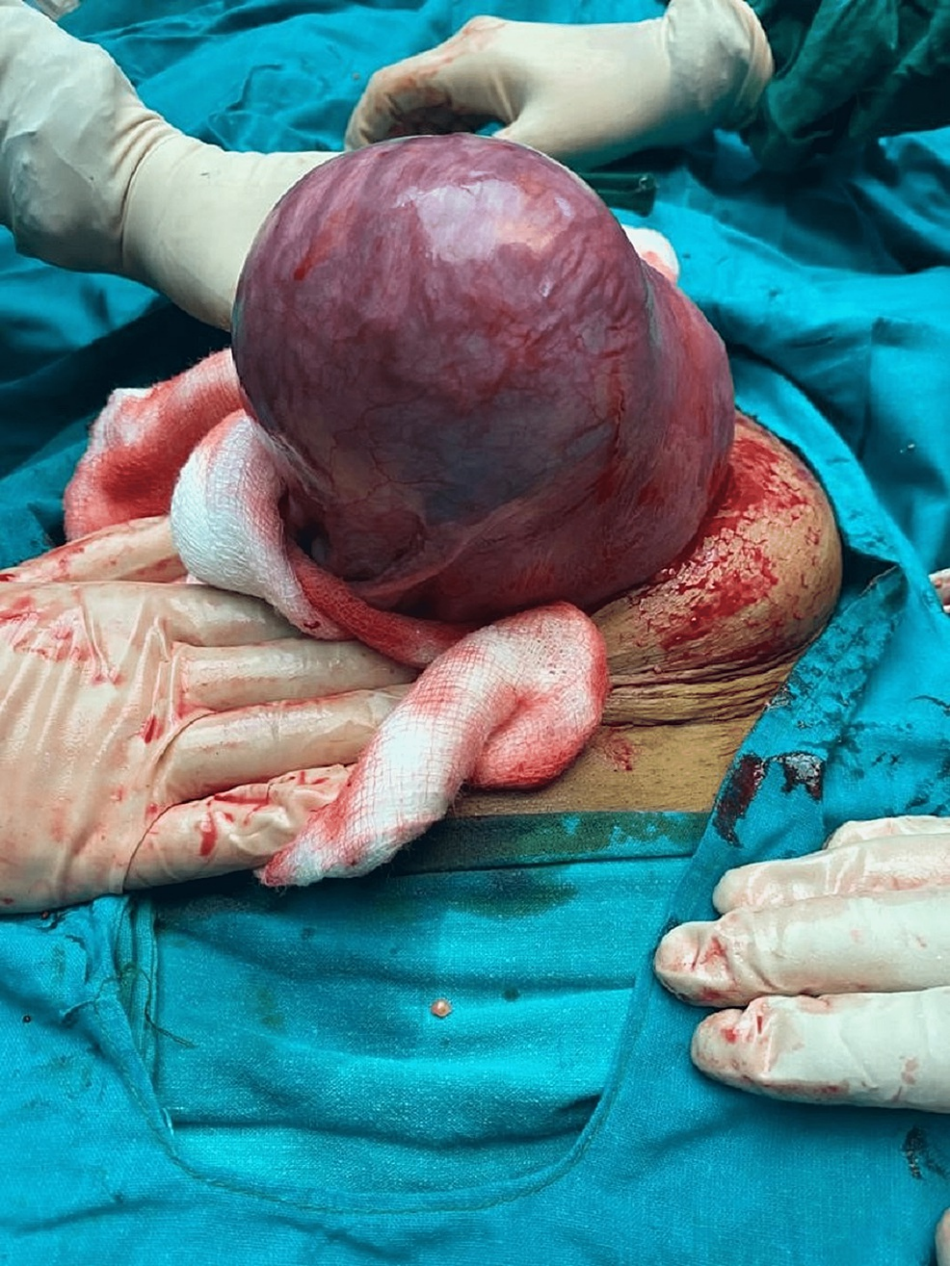
Right unicornuate uterus with a non-communicating right rudimentary horn (cephalic view)

**Figure 2:**
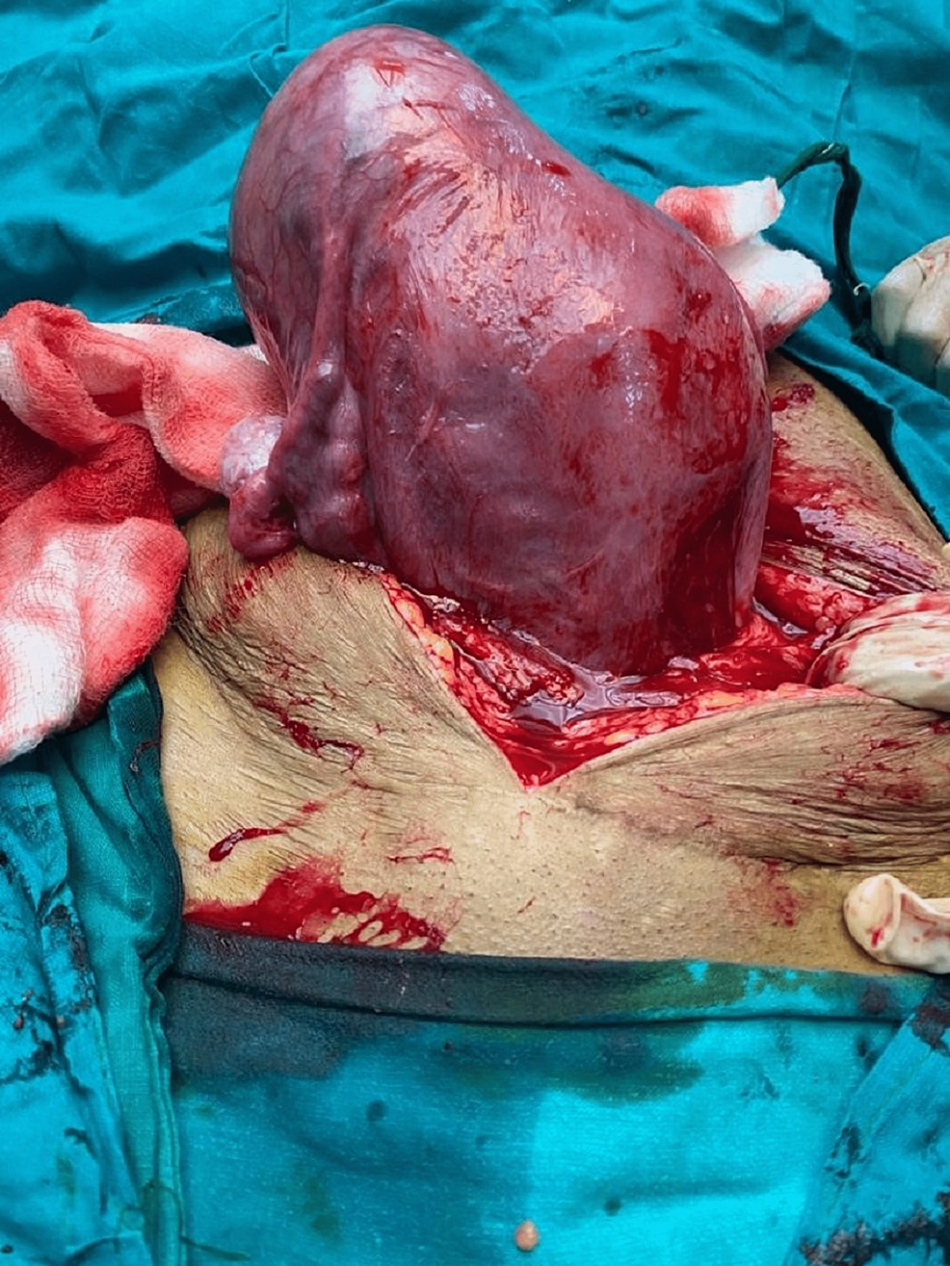
Right unicornuate uterus with a non-communicating rudimentary horn with the right fallopian tube and ovary (anterior view). Adnexal appendages (fallopian tube and ovary) are absent on the right side.

We present an exceptional pregnancy case of a unicornuate uterus and non-communicating rudimentary horn. A 28-year-old woman, gravida 2 para 1, was admitted to our hospital with abdominal pain and premature rupture of membranes (Figure 1 and Figure 2). She had a history of one previous normal vaginal delivery. This patient was not registered for prenatal care and presented to us at 37 weeks gestation. She had been married for three years in her medical history, and her menstrual cycles were regular with dysmenorrhea. No significant medical, familial, or psychological factors, including relevant genetic information. Both of her pregnancies occurred spontaneously.

During the clinical examination, the patient appeared pale, her pulse rate was 110 beats per minute, and her blood pressure was 100/70 mmHg. Generalized tenderness was noted in the abdomen, and the fundal height was measured at 36 weeks. Vaginal leakage was observed along with fetal bradycardia at 100 beats per minute. According to available medical records, her recent prenatal visits had been uneventful. She reported that her previous normal vaginal delivery occurred at a different hospital. Both pregnancies were spontaneous. Due to her arrival in the emergency department with premature rupture of membranes and fetal bradycardia, an emergency cesarean section was immediately arranged. The anesthetic and pediatric teams were notified accordingly. She experienced an uneventful pregnancy throughout her three trimesters and received regular prenatal care.

A Pfannenstiel's incision was performed, and a baby boy weighing 2900 grams (Apgar score = 8) was delivered in cephalic presentation, with the umbilical cord wrapped around the neck three times under spinal anesthesia. Upon inspection, the uterus was found to have a cylindrical shape with a flattened right-side wall and the absence of a right tubal ostium and tube. Tubal ligation was performed on the left side using the modified Pomeroy's method. The uterus was sutured in a double layer. The cesarean section proceeded without complications, and the patient was discharged after the routine 48-hour postoperative clinical follow-up period.

This case report emphasizes the possible hazards and obstacles linked with unicornuate pregnancy, such as fetal growth restriction and preterm labor. Early identification and meticulous monitoring play a vital role in effectively managing such pregnancies, aiming to maximize favorable outcomes for both the fetus and mother. Providing counseling on potential risks and ensuring regular antenatal care is crucial to facilitate timely interventions and the effective management of any complications.

## Discussion

Our case report presents an exceptional case of pregnancy in a unicornuate uterus with a non-communicating rudimentary horn. While unicornuate uterus and rudimentary horn pregnancies have been reported in the literature, each case has distinct characteristics and challenges.

The unicornuate uterus is a rare Mullerian anomaly, occurring in approximately 0.1% of the general population [5]. A non-communicating rudimentary horn adds to the case's complexity, as it signifies a complete absence of connection between the rudimentary horn and the main uterine cavity. Although isolated cases of pregnancy in the unicornuate uterus and rudimentary horns have been documented, the unique anatomical features observed in our patient distinguish it from previously reported cases.

Comparing our case to similar case reports, several common challenges and risks associated with unicornuate pregnancies emerge. Fetal growth restriction and preterm labor are frequently encountered in pregnancies with a unicornuate uterus [6-8]. These risks are attributed to the limited uterine expansion capacity and the developing fetus' altered blood supply. Our case aligned with these patterns, as the patient presented with premature rupture of membranes and required an emergency cesarean section due to concerns about fetal well-being.

In terms of management, early identification of the unicornuate uterus is crucial for initiating appropriate prenatal care and monitoring. Regular antenatal visits allow for the detection of any potential complications and the implementation of timely interventions. It is important to note that our patient did not receive prenatal care until her admission to the hospital, highlighting the significance of ensuring that all pregnant individuals have access to comprehensive prenatal services [9,10].

Counseling plays a pivotal role in the management of unicornuate pregnancies. Patients need to be informed about the potential risks associated with this condition, including the increased likelihood of adverse obstetric outcomes. They should also be educated about the importance of spacing pregnancies and employing effective contraception methods to optimize their reproductive health and minimize the potential risks of subsequent pregnancies [11,12]. In our case, the patient was counseled on contraception use for at least six months.

While our case report provides valuable insights into managing unicornuate pregnancies, it is important to acknowledge the limitations of this study. The report represents a single case, and generalizations should be made cautiously. Further research and more extensive case studies are necessary to enhance our understanding of the unique aspects and challenges associated with unicornuate uterus and rudimentary horn pregnancies [13,14].

## Conclusions

Congenital uterine malformations, including a unicornuate uterus with a rudimentary horn, are relatively uncommon but significant conditions that impact women's reproductive health. These abnormalities can lead to obstetrical risks, such as miscarriages, malpresentation, fetal growth restriction, preterm delivery, and rupture of the rudimentary horn. Accurate diagnosis and classification of these malformations are crucial for managing and counseling affected individuals. However, determining the exact incidence remains challenging due to normal reproduction with many subtypes and personal choices regarding childbearing. Advancements in diagnostic techniques are needed to improve our understanding and healthcare strategies for these conditions, ensuring the well-being of women affected by congenital uterine malformations.
